# Interleukin-4 modulates type I interferon to augment antitumor immunity

**DOI:** 10.1126/sciadv.adt3618

**Published:** 2025-05-14

**Authors:** Hannah V. Newnes, Jesse D. Armitage, Anthony C. Buzzai, Emma de Jong, Katherine M. Audsley, Samantha A. Barnes, Shamini Srinivasan, Michael Serralha, Vanessa S. Fear, Belinda B. Guo, Matt E. Jones, Alistair R. R. Forrest, Bree Foley, Phil K. Darcy, Paul A. Beavis, Anthony Bosco, Jason Waithman

**Affiliations:** ^1^ School of Biomedical Sciences, The University of Western Australia, Perth, Australia.; ^2^ The Kids Research Institute Australia, The University of Western Australia, Perth, Australia.; ^3^ Department of Dermatology, Otto-von-Guericke University, Magdeburg, Germany.; ^4^ Cancer Immunology Program, Peter MacCallum Cancer Centre, Melbourne, Australia.; ^5^ Sir Peter MacCallum Department of Oncology, The University of Melbourne, Melbourne, Australia.; ^6^ Harry Perkins Institute of Medical Research, QEII Medical Centre and Centre for Medical Research, The University of Western Australia, Perth, Australia.; ^7^ Department of Pathology, The University of Melbourne, Melbourne, Australia.; ^8^ Department of Immunology, Monash University, Melbourne, Australia.; ^9^ Department of Immunobiology, The University of Arizona, Tucson, AZ, USA.

## Abstract

Despite advances in immunotherapy, metastatic melanoma remains a considerable therapeutic challenge due to the complexity of the tumor microenvironment. Intratumoral type I interferon (IFN-I) has long been associated with improved clinical outcomes. However, several IFN-I subtypes can also paradoxically promote tumor growth in some contexts. We investigated this further by engineering murine B16 melanoma cells to overexpress various IFN-I subtypes, where a spectrum of outcomes was observed. Characterization of these tumors by RNA sequencing revealed a tumor immune phenotype, where potent IFN-I signaling concomitant with diminished type 2 inflammation failed to confer durable tumor control. T cell–mediated rejection of these tumors was restored by introducing interleukin-4 (IL-4) into the tumor microenvironment, either through ectopic expression or in a preclinical adoptive T cell therapy model. Collectively, our findings highlight the IFN-I/IL-4 axis in promoting antitumor immunity, which could be harnessed to target and stratify solid tumors that are nonresponsive to frontline therapies.

## Teaser

A therapeutic strategy that harnesses the IL-4/IFN-I axis to improve antitumor T cell immunity.

## INTRODUCTION

Therapeutic strategies that bolster antitumor immunity have revolutionized the treatment of advanced melanoma. However, durable responses to current immunotherapies are only observed in a fraction of patients and the challenge remains to improve outcomes in immunotherapy-resistant disease and against other solid cancers, highlighting the need to develop more tailored treatment options. The tumor microenvironment (TME) is a dynamic network of immune, stromal, and cancer cells tuned to resist antitumor immune responses. Current paradigms in the field describe immunologically “hot” tumors as those enriched in cytotoxic lymphocytes and type 1 inflammation, whereas type 2 inflammation is considered a characteristic of “cold” tumors (*1*, *2*). Immunologically cold tumors are abundant with tumor-promoting cell types including fibroblasts, M2-polarized macrophages, type 2 innate lymphoid cells (ILC2), CD4^+^ T helper 2 (T_H_2) cells, myeloid-derived suppressor cells (MDSCs), and regulatory T cells (*3*–*6*). Cold tumor signatures are a common predictor of poor responses to immunotherapies such as adoptive cell therapy (ACT) and immune checkpoint blockade (ICB) (*7*). Thus, developing new strategies that render tumors more permissive to immune attack remains a major focus of current research.

It is well known that type I interferons (IFN-Is) are potent modulators of antitumor immunity. IFN-I plays a critical role in remodeling the TME and is also a hallmark of immunologically hot tumors (*4*). As such, adjuvants that boost IFN-I such as STING agonists or recombinant IFN-I in combination with other immunotherapy regimens are being extensively investigated (*8*–*10*). Several recent studies have demonstrated that elevated tumor IFN-I contributes to chimeric antigen receptor (CAR) T cell dysfunction and ICB resistance (*9*, *11*, *12*), highlighting nuances of IFN-I signaling that are not well understood in the cancer context.

IFN-Is are a functionally diverse family of cytokines consisting of 16 subtypes. The plasticity of the IFN-I response is thought to be mediated by a variety of factors, including the differential affinity of these subtypes for cognate receptors, IFNAR1 and IFNAR2, as well as differential downstream activation of STAT and interferon regulator factor (IRF) complexes (*13*–*15*). We have previously shown that distinct IFN-I subtypes can elicit a spectrum of outcomes in a murine B16 melanoma model. Notably, whereas most subtypes such as IFNα4 could temporarily restrict tumor outgrowth, the IFNα9 subtype promotes immune-mediated tumor rejection, effectively curing a subset of mice from disease (*16*). In this study, we interrogated this model further using bulk and single-cell transcriptomics to unravel the underlying mechanisms of these divergent responses. Notably, molecular characterization of these tumors demonstrates that IFNα4 drives an intense yet ineffective IFN-I response that also ablates type 2 inflammatory cells in the TME, ultimately resulting in defective T cell immunity. Delivery of the canonical type 2 inflammatory cytokine, interleukin-4 (IL-4) into the TME could successfully restore tumor control in the presence of IFNα4 in a preclinical ACT model. This elucidates the role of type 2 inflammation in fine-tuning antitumor immunity. Critically, we observed similar tumor transcriptional signatures across a range of different solid cancers that associated with clinical outcomes. Collectively, these findings may guide the development of improved personalized cancer therapeutics that harness the IFN-I/IL-4 axis to reinvigorate antitumor T cell responses against solid tumors.

## RESULTS

### Stronger transcriptional IFN-I responses do not correlate with improved tumor clearance

**Fig. 1.**
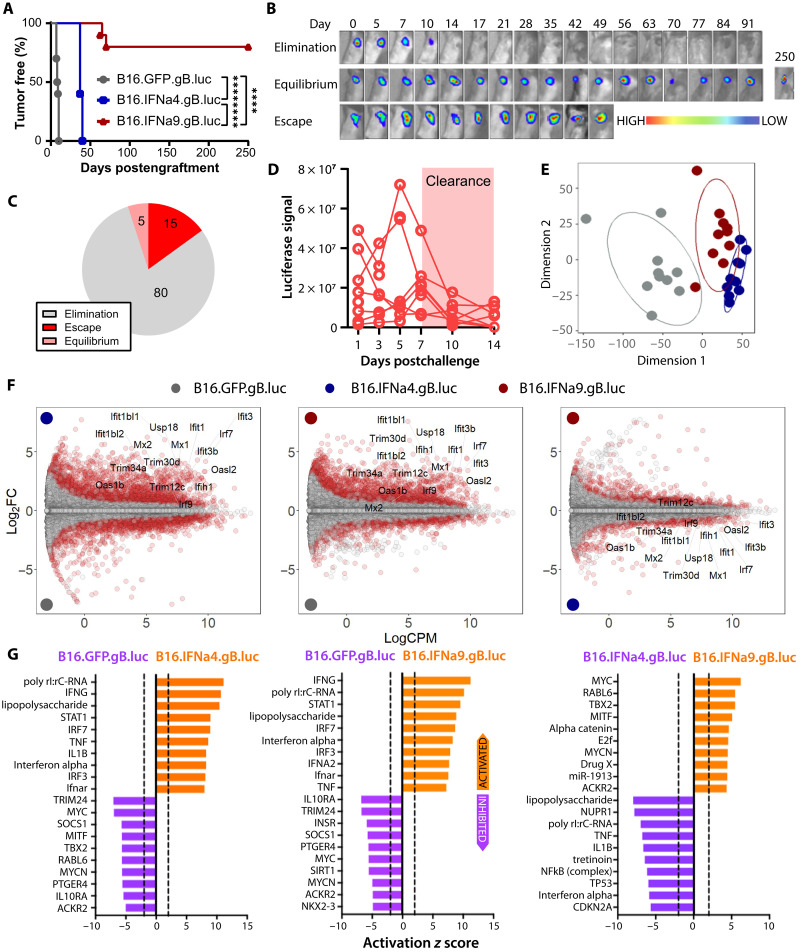
Survival and growth kinetics of IFN-I–secreting tumors and underlying bulk transcriptomic signatures. C57BL/6 mice were inoculated subcutaneously with 5 × 10^5^ B16.GFP.gB.luc, B16.IFNα4.gB.luc, or B16.IFNα9.gB.luc cells. (**A**) Kaplan-Meier plot representing the percentage of tumor-free mice after tumor challenge. Data were pooled from two to three independent experiments (*n* = 10 to 30 mice per group). (**B**) Representative images from IVIS bioluminescence longitudinal imaging of mice inoculated with B16.IFNα9.gB.luc cells. (**C**) Proportions of mice challenged with B16.IFNα9.gB.luc cells that underwent tumor elimination, equilibrium, or escape events. (**D**) Luciferase signal (photons/s) over time during tumor elimination. (**E**) Multidimensional plot of global gene expression data. (**F**) Smear plots illustrating gene expression differences between each tumor type. (**G**) Predicted upstream regulators driving transcriptional differences between tumor types. Survival data were analyzed by the log-rank Mantel-Cox test. ****P < 0.0001.

We have previously demonstrated that distinct IFNα subtypes secreted locally in the TME differ vastly in their capacity to control tumor growth (*16*). Here, we focus on two exemplary subtypes driving these disparate outcomes, IFNα4 and IFNα9. B16 melanoma cells were engineered to express IFNα4 or IFNα9 or empty vector control [green fluorescent protein (GFP)], the herpes simplex virus (HSV)–derived glycoprotein B (gB), and firefly luciferase (luc) (fig. S1). Inoculation of C57BL/6 mice with the empty vector control resulted in rapid exponential growth of tumors (Fig. 1A). In contrast, expression of IFNα4 or IFNα9 resulted in divergent phenotypes, ranging from an overall survival of 80% in the B16.IFNα9.gB.luc cohort to the development of chronic and persistent tumors, while significantly delayed, in all mice from the B16.IFNα4.gB.luc cohort. Longitudinal bioluminescent imaging was used to track disease progression in mice engrafted with B16.IFNα9.gB.luc cells (Fig. 1B). Complete control and elimination was observed in 80% of mice, whereas 15% of tumors escaped and progressed and 5% persisted for up to 250 days postengraftment (Fig. 1C). Moreover, successful elimination occurred within a 2-week period, whereby the bioluminescent signal began to regress after 7 days and was absent by day 14 (Fig. 1D). To determine whether the IFN-I activity acted on the immune system or alternatively the tumor cells and parenchyma, we made use of IFNAR^o/o^ mice that are refractory to IFN-I signaling (fig. S2). IFNAR^o/o^ mice were lethally irradiated and reconstituted with either wild-type (WT) bone marrow or IFNAR^o/o^ bone marrow. In addition, reverse chimeras were generated where irradiated WT mice are the recipients of donor bone marrow. After 8 weeks, to allow for complete reconstitution, chimeric mice were challenged with B16.IFNα9.gB.luc cells and monitored for tumor development. WT➔IFNAR^o/o^ chimeric mice, where only bone marrow–derived immune cells are responsive to IFN-I, were protected from tumor development at a similar rate as control WT➔WT chimeras. Whereas IFNAR^o/o^➔IFNAR^o/o^ and IFNAR^o/o^➔WT chimeras, where either no cells can respond to IFNα or only the parenchyma can respond to IFNα, respectively, all developed tumors. These results demonstrated that the antitumor effect of IFNα9 was dependent on signaling through the bone marrow–derived immune compartment, confirming immune-mediated tumor elimination.

Next, to identify the molecular mechanisms underlying IFN-I–mediated tumor control, we performed bulk RNA sequencing (RNA-seq) to elucidate tissue-level transcriptional differences between tumor types 8 days postinoculation (*n* = 10 per group). Overall, B16.GFP.gB.luc, B16.IFNα4.gB.luc, and B16.IFNα9.gB.luc tumors exhibit distinct gene expression profiles (Fig. 1E). Expectedly, expression of interferon-stimulated genes (ISGs) and predicted IFN-I–related upstream regulators [i.e., STAT1, IRF7, IFNα, and polyinosinic:polycytidylic acid (poly I:C)] was increased in IFN-I–secreting tumors compared to controls (Fig. 1, F and G). However, this response was significantly stronger in tumors exposed to IFNα4 compared to IFNα9. Collectively, these findings suggest that the lack of tumor control observed in B16.IFNα4.gB.luc tumors was not due to diminished IFN-I signaling but rather a more intense IFN-I response. However, we cannot exclude the possibility that IFNα4 versus IFNα9 have distinct effects in other cell types in the TME that are interacting with the tumors.

### Dysregulated IFN-I and type 2 inflammation is associated with poor tumor control

**Fig. 2.**
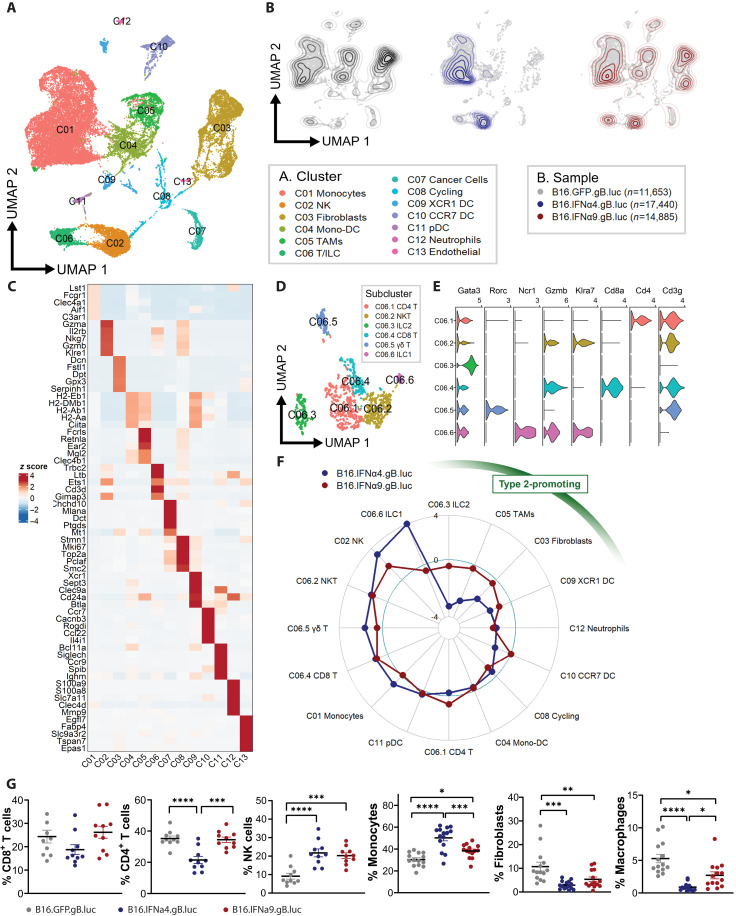
Single-cell transcriptomic characterization of B16.IFNα.gB.luc tumors. (**A**) Global UMAP plot annotated for immune, stromal, and cancer cell clusters. (**B**) Representative UMAP plots for B16.GFP.gB.luc, B16.IFNα4.gB.luc, and B16.IFNα9.gB.luc tumors. (**C**) Gene expression heatmap highlighting the top 5 marker genes per annotated cluster. (**D**) UMAP plot representing the C06 T/ILC cluster divided into six subclusters. (**E**) Violin plots of top marker genes associated with each subcluster. (**F**) Radar plot visualizing the fold change of immune and stromal population frequencies in B16.IFNα4.gB.luc and B16.IFNα9.gB.luc tumors, relative to B16.GFP.gB.luc tumors. (**G**) Flow cytometry validation of cell populations between the three tumor types. For both RNA-seq and flow cytometry experiments tumors were harvested 8 days post inoculation. Data were pooled from two to three independent experiments (*n* = 9 to 15 per group). One-way ANOVA with a Tukey post hoc test was used to measure differences in flow cytometry proportions. *P < 0.05; **P < 0.01; ***P < 0.001; ****P < 0.0001.

To obtain more detailed information about the cellular and molecular events underlying IFN-I–mediated tumor control, we performed single-cell RNA sequencing (scRNA-seq) to determine the differences between tumor types (*n* = 3 per group) (Fig. 2). Unsupervised clustering revealed 13 distinct cellular populations with unique transcriptional profiles, which were annotated by canvassing canonical marker gene expression for known cell compartments (Fig. 2, A to C). Secondary clustering of the C06 population (T cells and ILC) further revealed six subclusters demarcated by the expression of various T cell– and ILC-related genes (Fig. 2D). When compared to B16.GFP.gB.luc control tumors, we observed a reduction in type 2 inflammatory cells in B16.IFNα4.gB.luc tumors, most notably ILC2s, tumor-associated macrophages (TAMs) and fibroblasts (Fig. 2, E and F). These TAMs predominantly exhibited hallmarks of alternative activation (M2 polarized) based on the strong expression of canonical markers such as *Retnla*, *Mrc1*, and *Arg1* (data S1). Conversely, we observed an enrichment of innate lymphoid cells, such as natural killer (NK) cells in B16.IFNα4.gB.luc tumors. However, these cells expressed similar mRNA levels for various functional markers between conditions (fig. S3). Changes in cell type frequency were validated by multiparametric flow cytometry (Fig. 2G and fig. S4).

**Fig. 3.**
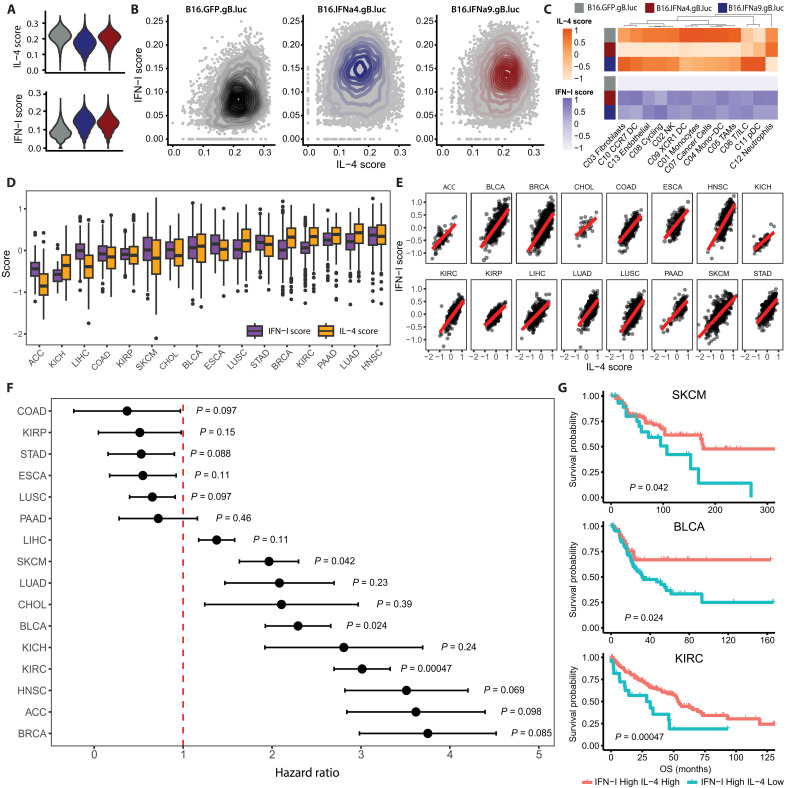
IFN-I-high and type 2-high inflammatory signature is positively associated with patient survival in multiple tumor entities. (**A**) Violin plot of IL-4 and IFN-I gene expression scores between B16.GFP.gB.luc, B16.IFNα4.gB.luc, and B16.IFNα9.gB.luc tumors. (**B**) Scatterplots visualizing the distribution of IL-4 and IFN-I gene expression scores (all cells) across each tumor type. (**C**) Heatmap visualization of IL-4 and IFN-I scores across each major cell compartment between B16.GFP.gB.luc, B16.IFNα4.gB.luc, and B16.IFNα9.gB.luc tumors. (**D**) Distribution of IFN-I and type 2 inflammation (T2) gene expression scores calculated across 16 different TCGA cancer types. (**E**) Correlations between IFN-I and T2 scores within cancer types. (**F**) Forest plots detailing hazard ratios (95% confidence interval) between IFN-I^High^T2^High^ and IFN-I^High^T2^Low^ patient groups. (**G**) Kaplan-Meier plots representing overall survival (OS) between IFN-I^High^T2^High^ and IFN-I^High^T2^Low^ patient groups for SKCM, BLCA, and KIRC cohorts.

We next evaluated whether these cell populations displayed intrinsic differences in both IFN-I and type 2 inflammatory signaling across the different models. IL-4 is one of the most potent drivers of type 2 inflammation. Analysis of receptor transcripts showed the highest expression of *Il4r* in these B16 tumors compared to other prominent type 2 effectors such as IL-5 (*Il5r*) and IL-13 (*Il13ra1* and *Il13ra2*) (fig. S5). Thus, we focused primarily on events downstream of IL-4 signaling. Gene expression scores computed for both IL-4 (GO:0071353) and IFN-I (GO:0071357) signaling highlighted an IFN-I–skewed response in B16.IFNα4.gB.luc tumors, whereas a balanced IFN-I/IL-4 response was observed in B16.IFNα9.gB.luc tumors (Fig. 3, A and B). These patterns were observed on a cluster-specific and a global level (Fig. 3C). We canvassed human clinical data [The Cancer Genome Atlas (TCGA) pan-cancer atlas] to determine whether this imbalanced signature could inform clinical outcomes. Gene expression scores were calculated across 16 different cancer types, revealing strong correlations between IFN-I and type 2 inflammatory responses (Fig. 3, D and E). Notably, patients presenting with IFN-I–skewed tumors with lower type 2 inflammatory signatures were associated with poor overall survival, particularly in solid cancers such as skin cutaneous melanoma (SKCM), bladder cancer (BLCA), and kidney renal clear cell carcinoma (KIRC) (Fig. 3, F and G).

### Endogenous CD8^+^ T cells are indispensable to the antitumor response but are impaired in the presence of IFNα4

**Fig. 4.**
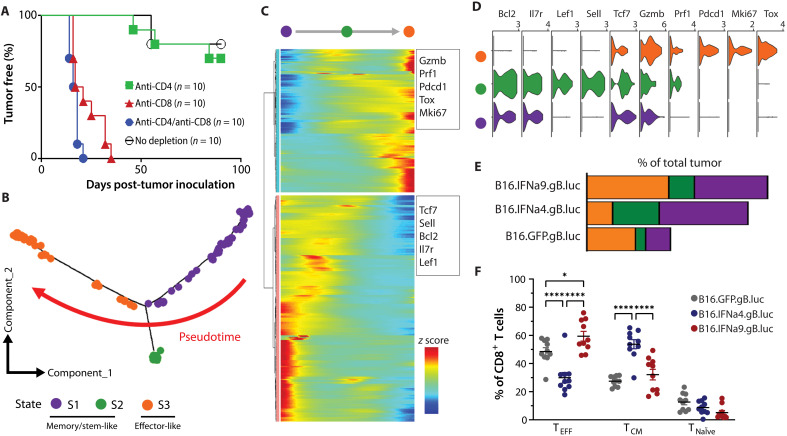
IFNα4 reduced tumor-infiltrating effector CD8^+^ T cells compared to IFNα9. (**A**) C57BL/6 mice were treated with either PBS, 100 μg of anti-CD4, 100 μg of anti-CD8, or 100 μg of both anti-CD4 and anti-CD8 weekly for 6 weeks. One day after the second depletion, mice were challenged with 5 × 10^5^ B16.IFNα9.gB.luc cells. Tumor development was measured over time. (**B**) Trajectory plot of endogenous CD8^+^ T cells from all tumors and their corresponding pseudotime state. (**C**) Pseudotime heatmap illustrating two distinct clusters of genes active at early versus late pseudotime. (**D**) Violin plots of canonical T cell gene markers associated with stemness/memory and effector function. (**E**) Distribution of CD8^+^ T cell states (scRNA-seq) across each tumor type. (**F**) Flow cytometry validation of CD8^+^ T cell populations between the three tumor types, effector (T_EFF_, CD44^+^), central memory (T_CM_, CD44^+^CD62L^+^), and naïve (T_naïve_, CD62L^+^). Data were pooled from two independent experiments (*n* = 10 per group). One-way ANOVA with a Tukey post hoc test was used to measure differences in flow cytometry proportions. *P < 0.05; ****P < 0.0001.

To determine the role of the endogenous T cell compartment in driving tumor clearance, we performed T cell depletion experiments. C57BL/6 mice were treated with αCD4 and αCD8 monoclonal antibodies prior to engraftment of B16.IFNα9.gB.luc tumors. Notably, only depletion of CD8^+^ T cells resulted in a loss of tumor control, suggesting their critical role in driving tumor elimination in the presence of IFNα9 (Fig. 4A). Pseudotemporal analysis of CD8^+^ T cells (cluster C06.4; Fig. 2D) revealed three transcriptional states (Fig. 4B), underscored by an enrichment of memory- and stemness-related transcripts (*Tcf7*, *Sell*, *Bcl2*, *Il7r*, and *Lef1*) and effector-related transcripts (*Gzmb*, *Prf1*, *Pdcd1*, *Tox*, and *Mki67*) at early (S1) versus late (S2 and S3) trajectories respectively (Fig. 4, B to D). These CD8^+^ T cells with elevated effector-related genes were threefold higher in B16.IFNα9.gB.luc tumors compared to B16.IFNα4.gB.luc tumors (Fig. 4E). These frequencies were confirmed by flow cytometry, highlighting an increase in CD62L^−^CD44^+^ (effector) CD8^+^ T cells in IFNα9-secreting tumors, compared to IFNα4-secreting counterparts (Fig. 4F).

### Overexpression of IL-4 can reinstate efficient tumor control in IFN-I^high^T2^low^ tumors

**Fig. 5.**
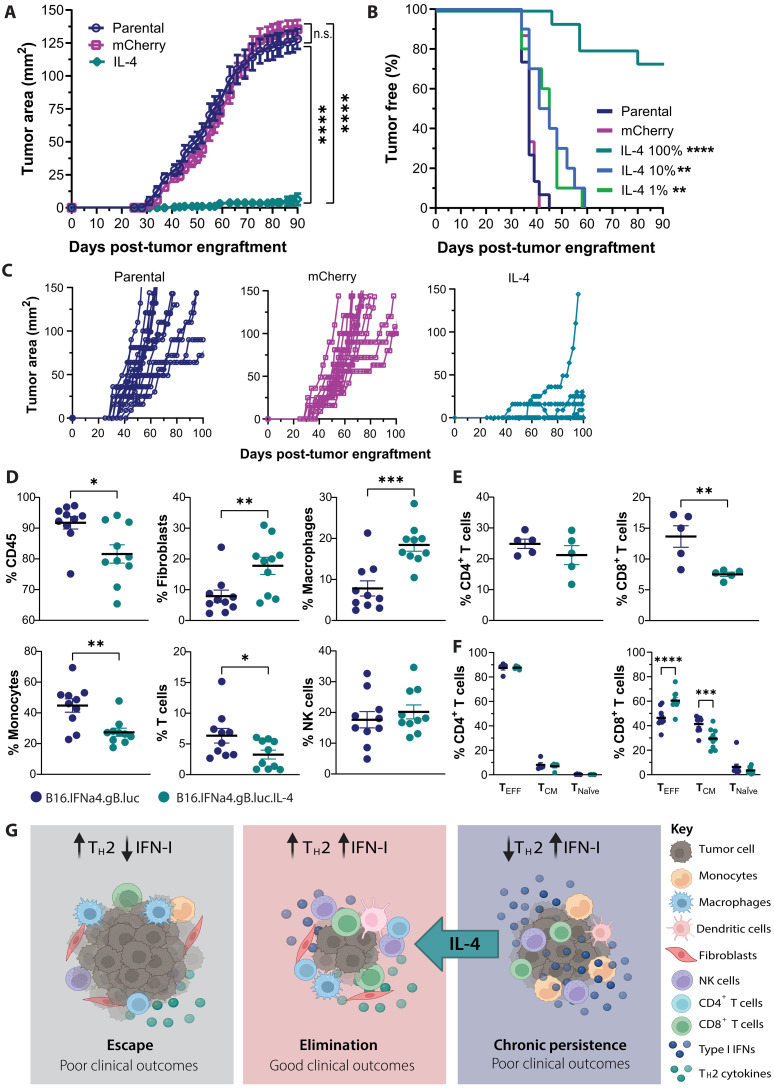
Tumor-derived IL-4 restores antitumor control in IFNα4-secreting tumors compared to control. C57BL/6 mice were inoculated subcutaneously with 5 × 10^5^ B16.IFNα4.gB.luc cells, B16.IFNα4.gB.luc.mCherry cells, or B16.IFNα4.gB.luc.IL4 cells or ratios 10 or 1% B16.IFNα4.gB.luc.IL-4 cells to B16.IFNα4.gB.luc cells. (**A**) Tumor growth was measured over time; data were pooled from three independent experiments (*n* = 15 mice per group). Data depict the means ± SEM. Repeated-measures two-way ANOVA (mixed-model) with a Tukey post hoc test was used to measure differences between groups. (**B**) Percentage of tumor-free mice after tumor challenge. Data were pooled from two to three independent experiments (*n* = 10 to 15 mice per group) and analyzed by the log-rank Mantel-Cox test comparing to the parental group. (**C**) Tumor growth measurements from individual mice. (**D** to **F**) Eight days after mice were challenged, tumors were harvested and analyzed by flow cytometry. Dots indicate biological samples, error bars show the SEM, and data were pooled from two independent experiments (*n* = 10 mice per group). (D) Percentage of CD45^+^ cells, fibroblasts, macrophages, monocytes, granulocytes, B cells, T cells, and NK cells of viable nonmalignant cells. (E) Percentage of CD4^+^ and CD8^+^ T cells of total CD3^+^ T cells, and data were analyzed by unpaired *t* tests. (F) Proportion of effector (T_EFF,_ CD44^+^), central memory (T_CM_, CD44^+^CD62L^+^), and naïve (T_naïve,_ CD62L^+^) CD8^+^ or CD4^+^ T cells, and data were analyzed by two-way ANOVA with a Šídák post hoc test. *P < 0.05; **P < 0.01; ***P < 0.001; ****P < 0.0001. n.s., not significant. (**G**) Schematic depicting three tumor phenotypes driven by IFN-I and type II inflammation in the TME. Created in BioRender. S.A.B. (2025); https://BioRender.com/e32i228.

The above data indicated that an imbalanced/IFN-I skewed TME may result in poor tumor control, suggesting the hypothesis that interventions that reinstate a balanced TME will potentially improve tumor control. To test this hypothesis, B16.IFNα4.gB.luc cells were transduced with retrovirus expressing *Il4* or empty vector (fig. S6). C57BL/6 mice were engrafted with either the parental B16.IFNα4.gB.luc (IFNα4_parental), B16.IFNα4.gB.luc.mCherry (IFNα4_mCh), or B16.IFNa4.gB.luc.Il4 (IFNα4_IL4). Notably, 70% of mice engrafted with IFNα4_IL4 were tumor free at day 90 whereas all mice engrafted with IFNα4_parental or IFNα4_mCh cells had developed palpable tumors by day 45 postengraftment (Fig. 5, A to C). Mice that did develop palpable tumors following engraftment with IFNα4_IL4 cells had tumors that were much smaller and grew significantly slower (Fig. 5A). Simultaneously, mice were engrafted with either 1 or 10% of cells expressing IL-4 and 99 and 90% of B16.IFNα4.gB.luc cells, respectively, had a significant increase in tumor free survival compared to controls.

To interrogate the immune response within the TME, we used flow cytometry 8 days postengraftment. We found a significant shift in multiple cell types within the TME including an increase in macrophages and fibroblasts and reduced proportions of monocytes (Fig. 5D). Analysis of the T cell compartment demonstrated that there were less overall CD8^+^ T cells in the TME; however, we observed a significant increase in effector (CD62L^−^CD44^+^) CD8^+^ T cells and reduction in central memory (CD44^+^CD62L^+^) CD8^+^ T cells (Fig. 5, E and F). This suggests that the presence of IL-4 modulates the cellular composition of the TME and, critically, the CD8^+^ T cell compartment, rescuing the effector phenotype.

### T cells delivering IL-4 can reduce tumor burden in preclinical models of IFN-I^high^T2^low^ tumors

**Fig. 6.**
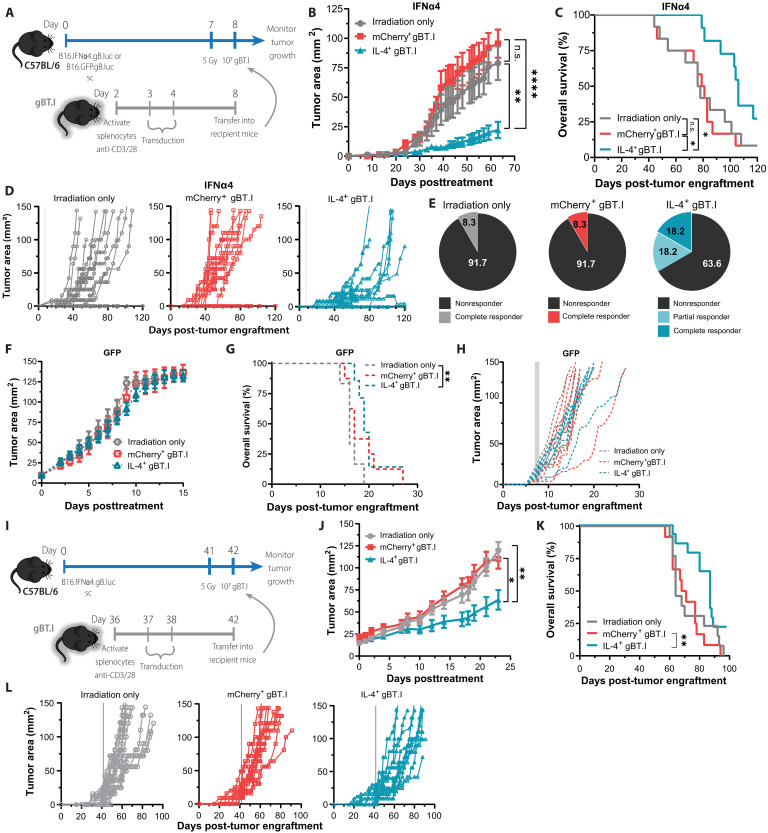
ACT with T cell–secreting IL-4 reduces tumor burden and improves survival in IFN-I-high tumors. (**A** and **I**) Schematic outlining the experimental timelines. C57BL/6 mice were challenged with 5 × 10^5^ B16.IFNα4.gB.luc (**B** to **E** and **J** to **L**) or B16.GFP.gB.luc cells (**F** to **H**); 7 days later, mice were irradiated (500 rad) and randomized into three treatment groups. Mice either received no further treatment, 10^7^ mCherry^+^ gBT.I cells, or 10^7^ IL-4^+^ gBT.I cells. Gy, gray; sc, subcutaneously. [(B), (F), and (J)] Tumor growth was measured over time; each point represents the means ± SEM from three independent experiments (*n* = 11 to 12 per group). [(C), (G), and (K)] Kaplan-Meier plots representing the overall survival of mice post tumor inoculation. [(D), (H), and (L)] Tumor growth measurements of individual mice. (E) Proportion of nonresponders; mice that were culled due to tumor endpoint, partial responders; mice with tumors less than maximum size (150 mm^2^) after 120 days or complete responders; mice that had no tumor after 120 days. Data were analyzed by repeated-measures two-way ANOVA (mixed-model) followed by a Tukey post hoc test [(B) and (G)] or log-rank Mantel-Cox test [(C) and (H)]. *P < 0.05; **P < 0.01; ****P < 0.0001.

We next sought to interrogate the therapeutic potential of IL-4 in the IFN-I^high^T2^low^ TME via an ACT model. First, we tested this strategy in an early model of disease progression involving treatment 7 days post-tumor engraftment of C57BL/6 mice. Mice received lymphodepletion followed by adoptive transfer of transgenic CD8^+^ T cells, which express a T cell receptor (TCR) specific for the HSV immunodominant gB_498-505_ peptide (gBT.Is) (*17*). Transferred gBT.I cells were retrovirally transduced to express IL-4 or mCherry vector control (Fig. 6A and fig. S7). Mice treated with IL-4–producing gBT.Is (IL-4^+^ gBT.I) had significantly smaller tumors after 70 days (Fig. 6B). Clearly, there was no therapeutic effect when treated with empty vector tumor-specific T cells (mCherry^+^ gBT.Is). Critically, there was also a significant increase in overall survival in mice treated with IL-4^+^ gBT.Is as compared to irradiation only or mCherry^+^ gBT.Is (Fig. 6C). Moreover, over a third of mice treated with IL-4^+^ gBT.I cells were either complete or partial responders 120 days after tumor engraftment (Fig. 6E). In addition, when this ACT protocol was tested in mice bearing B16.GFP.gB.luc (IFN-I^low^) tumors, we saw no impact on tumor growth and a significant but moderate increase in overall survival in mice treated with IL-4^+^ gBT.I cells (19 days) compared to irradiation (16 days) but not mCherry^+^ gBT.Is (17 days) (Fig. 6, F to H).

This treatment protocol was next tested in an advanced model of disease, whereby 41 days after tumor engraftment mice underwent the same treatment protocol (Fig. 6I). Again, there was a significant reduction in tumor growth in mice treated with IL-4^+^ gBT.Is but not in mice treated with irradiation only or mCherry^+^ gBT.Is (Fig. 6J). In addition, treatment with IL-4^+^ gBT.Is saw a significant increase in overall survival (Fig. 6, K and L). Overall, this shows the therapeutic potential of using IL-4 in combination with ACT to make an IFN-I^high^T2^low^ TME more permissive to elimination.

## DISCUSSION

The use of IFN-I as an immune adjuvant for cancer therapy has gained considerable traction in recent years. However, the presence of tumor IFN-I often fails to correlate with improved antitumor immune responses or clinical outcomes. In this study, we sought to investigate the underlying mechanisms of antitumor immunity driven by specific IFN-I subtypes. We found a tumor immune phenotype characterized by intense IFN-I activation and ablated T2 inflammation. The presence of this transcriptional signature, compared to those with higher T2 inflammation in clinical samples was associated with improved outcomes across several solid cancer types. These important findings could improve the stratification of patients for therapy. We also demonstrate that IL-4 can be used therapeutically to target these tumors in preclinical models, highlighting a key strategy harnessing T2 inflammation to bolster antitumor immunity.

It has long been recognized that hot tumors with more IFN-I are generally more sensitive to treatment (*18*–*20*). However, recent studies have highlighted that a highly inflammatory and IFN-I–skewed TME showed resistance to therapy and worse patient outcomes (*21*, *22*). The dual role of IFN-I has been well reported in chronic viral settings, whereby IFN-I induces a negative feedback loop and consequent immunosuppression (*23*–*25*). Specific examples have shown that IFN-I can up-regulate the expression of checkpoint inhibitors to attenuate the antitumor T cell response (*11*, *26*). Thus, like chronic viral infections, persistent IFN-I signaling in cancer could be a key driver of immune dysfunction, highlighting the need to optimize treatment strategies that can effectively fine-tune this axis. Our data support this notion, given that dysregulated CD8^+^ T cell responses were associated with intense tumor IFN-I and an absence of key type 2 inflammatory cells, namely, TAMs (which predominantly exhibited an M2 phenotype), fibroblasts, and ILC2s. We describe the imbalance between tumor IFN-I and type 2 inflammation, and our findings may have notable clinical implications across the solid tumor landscape.

T_H_2 cytokines such as IL-4 and IL-13 drive the polarization of M2-like macrophages. Within the TME, these cells are known to promote angiogenesis and secretion of immunosuppressive factors, such as TGFβ (transforming growth factor–β), Arg1, and iNOS (inducible nitric oxide synthase), that, in turn, inhibit cytotoxic CD8^+^ T cell function (*27*). Similarly, cancer-associated fibroblasts have been shown to reprogram the TME through secretion of extracellular matrix (ECM) components and angiogenic and growth factors (*28*). Grauel *et al.* (*29*) showed that in vivo blockade of TGFβ could attenuate fibroblast activity in the TME to increase responsiveness to ICB. Chen *et al.* (*30*) also showed that targeting T_H_2-mediated immunity, through a T_H_2 cytokine inhibitor (suplatast tosilate), promoted a T_H_1-biased TME that synergized with ICB in preclinical models of breast cancer. Despite the well-known role of T_H_2 modulators in supporting the polarization of tumor-promoting cell types, numerous recent studies have shown that T_H_2 modulators can improve the response to therapy (*31*–*34*). Blomberg *et al.* (*31*) demonstrated that T_H_2-polarized CD4^+^ T cells producing IL-5 were critical in enhancing the response to ICB in breast cancer. Another recent study showed that IL-33, an alarmin with a key role in type 2 inflammation, promoted the expansion of stem-like CD8^+^ T cells in chronic viral infections. Moreover, IL-33 balanced the effect of IFN-I to sustain the pool of stem-like Tcf1^+^ T cells (*33*).

Our analysis of human data suggests that this balance between tumor IFN-I and type 2 inflammation may also have clinical ramifications. One notable observation was that IFN-I and type 2 inflammatory gene signatures in the TCGA datasets appeared to strongly correlate with one another. Evidence of this counter-regulation has been demonstrated by Kim and Lee (*35*), where activity of IL-4 and IFNα exert antagonistic effects on one another through the formation of pSTAT6 and pSTAT2 complexes, respectively. Given an imbalance of these signals (IFN-I^high^T2^low^) was shown to drive immune dysfunction in our murine models, we postulated that this phenotype may also be an important predictor of clinical outcomes. We found that the stratification of IFN-I^high^T2^low^ tumors was associated with poor clinical outcomes across a range of solid cancers, including melanoma. This finding gives precedence to a molecular subtype of tumors that respond poorly to therapy despite exhibiting strong IFN-I activity. As such, treating this subtype may require different interventions, such as type 2 inflammatory modulators. Of note, we also observed that tumors with higher intrinsic immunogenicity appear to have stronger differences in overall survival following stratification, suggesting that existing tumor immune activity may be a prerequisite for interventions combining IFN-I and type 2 inflammatory modifiers.

We demonstrate that the use of IL-4^+^ tumor-specific T cells in a murine ACT model is an effective means of targeting this tumor subtype. Consistent with this, a previous work has demonstrated that the adoptive transfer of T_H_2 cells successfully eradicates myeloma cells, although this effect was independent of CD8^+^ T cell activity (*36*). In addition, IL-4 secretion by T follicular helper (T_FH_) cells following anti–PD-1 therapy improved anticancer CD8^+^ T cell responses in draining lymph nodes (*34*). Despite these findings, it is important to note that IL-4 can also exert protumor effects. Maier *et al.* (*37*) reported that secretion of IL-4 by mregDC impaired antitumor T cell function, which was reversed upon IL-4 blockade. This work has since progressed to a phase 1b clinical trial, where near-complete clinical responses were observed in one of six patients with non-small cell lung cancer (NSCLC) using dupilumab (anti–IL-4Rα) in combination with PD-1/PD-L1 checkpoint inhibitors (*38*). This was associated with the induction of type 1 tumor inflammation and IFN-I. These conflicting results suggest that IL-4 is highly pleiotropic; however, bolstering type 2 inflammation using IL-4 in tumors with this imbalanced phenotype (IFN-I^high^T2^low^) may offer clinical benefits in this subset of patients.

There are limitations to our study. Although we have described tumor phenotypes where durable control can be achieved with a combination of IL-4/IFN-I, the underlying mechanisms of this response are yet to be fully elucidated. Although we could not detect IL-4 mRNA in our data, the loss of ILC2s in IFN^high^T2^low^ tumors suggests that these cells may be a critical source of tumor IL-4 in our model. It is also known that IL-4 can signal through two receptor complexes, IL-4Rα-IL-2Rγc (type 1 receptor) and IL-4Rα -IL-13Rα1 (type 2 receptor). These, in turn, can trigger different downstream signaling cascades (*39*). We noted that IFN-I did increase *Il2rg* gene expression (IL-2Rγc) across multiple cell types in our model, suggesting that a productive antitumor immune response in the presence of IFN-I/IL-4 may be orchestrated by the utilization of both type 1 and type 2 IL-4R complexes.

This study primarily focused on one model of B16 melanoma in C57BL/6 mice where we were able to extrapolate that the IFN-I/IL-4 axis was correlated with outcomes in patients across multiple cancer types with TCGA data. Recent publication by Feng *et al.* (*40*) corroborates our findings, by demonstrating that the treatment with IL-4 improved both ACT and ICB in multiple murine tumor models including two models of murine melanoma, B16F10 and YUMM1.7, and a murine colon adenocarcinoma model, MC38. Moreover, the authors showed that IL-4 could rescue exhausted CD8^+^ T cells and enrich the effector population by altering their metabolism and promoting glycolysis. In our study, we monitored tumor-specific T cell responses using a transgenic animal model expressing the immunogenic gB antigen. Previously, we showed that, even in the absence of this xenoprotein, we could still observe complete tumor control in 33% of mice engrafted with IFNα9 expressing tumors and delayed tumor progression in those expressing IFNα4.

To reinforce the clinical potential of our findings, more clinically relevant ACT models, such as using human CAR T cells, will need to be evaluated in future work. Bai *et al.* (*41*) have recently shown that type II immunity was key to CAR T cell persistence in patients with acute lymphoblastic leukemia and long-term remission. Moreover, they were able to recapitulate these findings in patient CAR T cells samples with the addition of IL-4. Collectively, these recent studies show the clinical potential of using IL-4 to overcome current barriers to treatment. Future studies will need to assess whether there is redundancy in the system and interrogate whether other T_H_2 modulators including the IL-33/ILC2 pathway can similarly augment the IFN-I^high^ TME.

In summary, we report a notable IFN-I^high^T2^low^ tumor phenotype that impairs antitumor T cell control and is associated with worse clinical outcomes. We demonstrate that the adoptive transfer of IL-4^+^ tumor-specific T cells can successfully target this phenotype. These findings may guide the development of new immunotherapeutics that harness type 2 inflammation as a tailored approach to treating patients with dysfunctional IFN-I activity.

## MATERIALS AND METHODS

### Experimental design

The objective of this study was to interrogate the role of IFN-I subtypes on tumor elimination. We used a controlled laboratory experiment using preclinical mouse models. We phenotyped ex vivo tumors using flow cytometry and RNA-seq. We then went on to investigate the effect of overexpressing IFNα subtypes and IL-4 in murine melanoma cell lines on tumor growth using retroviral systems. Last, we assessed whether IL-4–secreting tumor-specific T cells could similarly reduce tumor growth in IFN^hi^T2^low^ tumors and increase overall survival in vivo. Sample randomization and blinding were used for these studies where appropriate.

All animal studies were performed according ARRIVE guidelines and were approved by the Telethon Kids Institute and Harry Perkins Institute of Medical Research Animal Ethics Committee. All experiments were randomized and blinded where possible. For in vivo experiments, sample sizes were prospectively determined using G*Power software (v3.1.9.7) to estimate the required sample size based on a repeated-measures analysis of variance (ANOVA) with *a* = 0.05 and power (1 − *b*) = 0.95 to ensure adequate statistical power. Endpoints for in vivo experiments were prospectively selected. All experiments performed had at least two biological repeats, with each replicate consisting of at least two technical repeats. Detailed methods for in vitro and in vivo experiments, including cell numbers, treatment, and statistical tests, are described below and in each figure legend as appropriate.

### Cell lines

B16 murine melanoma cells were purchased from the American Type Culture Collection and routinely passaged and cultured at 70 to 80% confluency in RPMI media (Thermo Fisher Scientific) supplemented with 10% fetal calf serum (FCS) (Sigma-Aldrich), 2 mM l-glutamine, 50 μM 2-mercaptoethanol, streptomycin (100 μg/ml), and penicillin (100 U/ml) (all Thermo Fisher Scientific) (R10 media) at 37°C and 5% CO_2_. Human embryonic kidney (HEK) 293T and L929 cells were similarly passaged in Dulbecco’s modified Eagle’s medium (Thermo Fisher Scientific) supplemented with 10% FCS, 2 mM l-glutamine, 50 μM 2-mercaptoethanol, streptomycin (100 μg/ml), and penicillin (100 U/ml) (D10 media) at 37°C and 5% CO_2_.

### Mice

C57BL/6 female mice were purchased from the Animal Resources Centre, Murdoch, Western Australia. gB-specific TCR transgenic (gBT.I) mice were bred at the Harry Perkins Institute of Medical Research. Mice were typically used at 8 to 12 weeks. Animals were housed under pathogen-free conditions, and all studies were approved by the Telethon Kids Institute Animal Ethics Committee (AEC 252, 254, 269, 289, 325, 366, 388, and 392) or Harry Perkins Institute of Medical Research AEC (AE293).

### Tumor challenge

Mice were injected subcutaneously with 5 × 10^5^ cells in 50 μl of RPMI media. Tumor size was monitored using calipers, and tumor area was calculated using the following formula: length (mm) by width (mm). Mice with tumors > 150 mm^2^ were euthanized. Tumor-free mice were defined as mice with no palpable masses.

### Bioluminescence imaging

Mice inoculated with B16.IFNα9.gB.luc tumors were intraperitoneally injected with 200 μl of d-luciferin [15 mg/ml in 1x phosphate-buffered saline (PBS)] (PerkinElmer, USA). Mice were anesthetized 5 min after injection by inhalation isoflurane. While maintained under anesthetic, mice were imagined using the in vivo imaging system, IVIS Spectrum (PerkinElmer, USA). Images were acquired using a 1-min exposure time. Living Image software version 3.2 (PerkinElmer, USA) was used to quantitate the bioluminescent signal of the tumors.

### In vivo depletions

For CD8^+^ T cell and CD4^+^ T cell depletion experiments, mice received control PBS or 100 μg of anti-CD8a (YTS169) and/or 100 μg of anti-CD4 (GK1.5) 1 day before tumor challenge and at weekly intervals post-tumor inoculation.

### Flow cytometry analysis of ex vivo tumors

Tumors were harvested and diced using a scalpel before being resuspended in RPMI supplemented with collagenase III (3 mg/ml; Worthington Biochemical, USA) and DNAse I (5 μg/ml; Sigma-Aldrich, USA) for 30 min at 37°C. Samples were neutralized in R10. Resulting single-cell suspensions were passed through a 100-μm nylon filter and centrifuged before being lastly resuspended in fluorescence-activated cell sorting (FACS) buffer [1x PBS, 1% bovine serum albumin (Bovagen, USA), and 5 mM EDTA]. Single-cell suspensions were stained with monoclonal antibodies specific for mouse. Following surface stain, samples were fixed with 4% paraformaldehyde prior to permeabilization with Permeabilization Buffer (eBioscience) and stained with monoclonal antibodies. Cells were stained with Fixable Viability Stain 575V at 1:5000 (BD Biosciences, 565694) prior to surface staining and followed by FcR (Fc receptor) block at 1:100 (BD Biosciences, 553141). Cells were analyzed using the BD LSRFortessa and FlowJo software (BD Biosciences/TreeStar). The following anti-mouse FACS antibodies were used in this study: CD45 (clone 30-F11, BD Biosciences, 1:100, 561037), CD3 (clone 145-2C11, BD Biosciences, 1:200, 612803), CD19 (clone 1d3, BD Biosciences, 1:400, 562291), NK1.1 (clone PK136, BD Biosciences, 1:200, 569716), Ly6G (1A8, BD Biosciences, 1:200, 562700), CD90.2 (clone 30-H12, BD Biosciences, 1:200, 553012), I^A^/I^E^ (clone M5/114.15.2, BD Biosciences, 1:400, 566086), CD11b (clone M1/70, BD Biosciences, 1:200, 563553), CD68 (clone FA/11, BD Biosciences, 1:100, 566388), CD140a (clone APA5, BD Biosciences, 1:200, 742176), Ly6C (clone AL-21, BD Biosciences, 1:200, 560593), F4/80 (clone T45-23-42, BD Biosciences, 1:200, 565613), CD8a (clone 53-6.7, BD Biosciences, 1:200, 563786), CD4 (clone RM4-5, BD Biosciences, 1:400, 565634), CD44 (clone IM7, BD Biosciences, 1:400, 560569), and CD62L (clone MEL-14, BD Biosciences, 1:400, 565261).

### Plasmid constructs and transduction of B16 cells

B16-F10 cells were sequentially transduced, as previously described (*16*), with retroviral vectors containing (i) either murine IFNα4 or IFNα9 and enhanced GFP, (ii) a full-length membrane-bound form of HSV gB and cyan fluorescent protein (CFP), (iii) luc and puromycin resistance, and (iv) murine IL-4 (Il4) and mCherry to generate B16.GFP.gB.luc, B16.IFNα4.gB.luc, and B16.IFNα9.gB.luc and B16.IFNα4.gB.luc.Il4 and B16.IFNα4.gB.luc.mCh cell lines. Briefly, retroviruses were generated by transfecting HEK293T cell lines with (i) pMIG-IFNα, (ii) pMIC-gB, (iii) pacluc2, and (iv) pMICh-Il4, pMD.old.gag.pol, and pCAG-VSVG (*42*). B16 cells were next transduced with filtered retrovirus supernatant in the presence of polybrene (8 μg/ml; Sigma-Aldrich). Cell lines were subsequently sorted for GFP^+^ CFP^+^ cells using a BD FACSAriaIII cell sorter (BD Biosciences) to select stable B16.GFP/IFNα4/IFNα9.gB cell lines. GFP and CFP expression of sorted cell lines was confirmed using a BD LSRFortessa X-20 (BD Biosciences). Selection of luciferase-positive cells was done by culturing transduced B16 cells lines with puromycin (2 μg/ml)–containing media. Cell lines were lastly sorted for mCherry^+^ cells using a BD FACSAriaIII cell sorter (BD Biosciences) to select stable B16.IFNα4.gB.luc.mCh and B16.IFNα4.gB.luc.Il4 cell lines.

### gBT.I cell transduction and adoptive transfer

Primary gBT.I cells were activated and transduced as previously described (*16*). Briefly, cells were activated for 24 hours in R10 media supplemented with 10 mM Hepes, 1 mM sodium pyruvate (Thermo Fisher Scientific) (T cell media), anti-CD3 (0.5 μg/ml; clone 17A2, 562163), anti-CD28 (0.5 μg/ml; clone 37.51, 553294) (BD Biosciences), IL-2 (100 U/ml), and IL-7 (2 ng/ml; PeproTech). The following day, cells were purified by a Lymphoprep density gradient and then transduced with retroviral supernatant using spinfection for 90 min at 2000*g* in RetroNectin (Takara Bio)–coated plates. Briefly, retroviruses were generated by transfecting HEK293T cell lines with either pMICh or pMICh-Il4, pMD.old.gag.pol, and pCAG-eco (*42*). Spinfection was repeated the next day. Following transduction, gBT.I cells were expanded in T cell media with IL-7 (2 ng/ml) and IL-15 (10 ng/ml; PeproTech) for 4 days. Following expansion, 10^7^ transduced gBT.I cells were intravenously transferred into irradiated (500 rad) recipients that were challenged either 7 or 41 days prior with 5 × 10^5^ B16.IFNα4.gB.luc cells.

### Bulk RNA-seq

Total RNA was extracted from whole tumors using TRIzol (Invitrogen, Waltham, USA) and purified using the RNEasy MinElute Kit (Qiagen, Hilden, Germany). Sequencing libraries were prepared using the TruSeq Stranded mRNA Sample Prep Kit, and 50–base pair single-end read sequencing was performed using the Illumina HiSeq 2000 system (Illumina, San Diego, USA) according to the manufacturer’s instructions. Read quality was assessed using FastQC (v0.11.3) followed by alignment to the mouse genome (mm11 reference) using HISAT2 (v2.0.4). Gene-level quantitation of aligned reads was calculated using SummerizeOverlaps (v1.7.2) and SAMStat (v1.5.2) used to perform postalignment quality control. For downstream analysis, differentially expressed genes (DEGs) were calculated using DESeq2 (v3.19) and using a negative binomial distribution model, with Benjamini-Hochberg–adjusted correction of *P* values for multiple comparisons. Principal components analysis was performed using prcomp() within R on all genes with an average expression of 10 counts per sample. Upstream regulator analysis (URA) was performed using the Ingenuity Pathway Analysis software (Qiagen, Hilden, Germany) to identify putative drivers of gene expression changes between groups.

### Single-cell RNA-seq

#### Preprocessing and quality control

Single-cell suspensions from digested tumors were processed with V3 chemistry using the Chromium Next GEM Single Cell 3’ Gel Bead Kit v3.1 (16 rxns, PN-1000122, 10X Genomics) Chip G and Chromium Next GEM Single Cell Library Prep Kit (16 rxns, PN-1000190, 10X Genomics), according to the manufacturer’s protocol. Samples were sequenced on the NovaSeq 6000 platform in a single batch. Raw FASTQ files were processed using CellRanger (Version 3.0.2, 10x Genomics) and aligned to the mouse GRCm38 genome assembly. The CellRanger count pipeline was run with default parameters. Count matrices and corresponding barcode and feature files were imported into the R statistical environment (version 3.6.2), and all subsequent quality control/analysis was conducted in R. Cells with <250 unique genes, <500 counts, and >12.5% mitochondrial content were excluded from further analysis.

#### Integration and clustering

Individual samples were integrated and analyzed further using Seurat (v3.2). Filtered data were normalized and log-transformed, and feature selection was performed to identify the top 2000 most variable genes. Integration anchors were identified from these top variable features using the FindIntegrationAnchors function, which performs canonical correlation analysis to align shared sources of variation across samples. The IntegrateData function was then used to harmonize the dataset, leveraging the identified integration anchors to align samples into a shared low-dimensional space. Uniform manifold approximation and projection (UMAP) was performed using the top 20 principal components as input variables, following by cluster partitioning using the Louvain algorithm at a resolution of 0.5. For T/NK subclustering, the “C06” cluster was extracted from the original dataset, and a separate UMAP was calculated using the top 250 variable features and top 20 principal components specific to that cluster. Louvain clustering was performed at a resolution of 0.25. Cluster annotation was assisted by determining canonical cell type marker gene expression, in addition to calculating DEGs for each cluster using a nonparametric Wilcoxon rank sum test with a Bonferroni adjustment for multiple comparisons.

#### Trajectory analysis

Cluster C06.4 (CD8^+^ T cells) was subset from the original analysis, and Monocle2 (v.2.4.0) was used to infer cell state transitions and pseudotime ordering on this population. Size factors for normalization were estimated with the estimateSizeFactors function, and dispersions necessary for differential expression testing were estimated using estimateDispersions. Dimensionality reduction was performed via the reduceDimension function using the DDRTree method. Cells were then ordered along the inferred trajectory using the orderCells function, with the root state set to cluster S1. A likelihood ratio test (with a Benjamini-Hochberg correction for multiple comparisons) was performed to infer genes that significantly change over pseudotime. The top 100 most significant genes were visualized by the plot_pseudotime_heatmap function.

### TCGA analysis

Gene expression and clinical data archived in the TCGA database were downloaded from the cBioPortal web platform (*n* = 16 cancer types). Batch-corrected, RNA-Seq by Expectation-Maximization (RSEM) count data were log-normalized and *z*-transformed. IFN-I and T2 inflammation gene expression scores were calculated by averaging the expression levels of genes in each respective pathway (GO:0034340 and GO:0002830 gene lists) in each sample. Survival analysis was performed across all cancer types. Using the survminer package (v0.4.9), IFN-I and T2 gene signatures were separately categorized into high and low groupings based on optimal cutpoints derived from the overall survival data (surv_cutpoint function). These were then combined to create four groupings based on high versus low, T2 and IFN-I gene signature scores. Cox proportional hazards regression was then performed to compare significant changes in overall survival between IFN^hi^T2^low^ and IFN^hi^T2^hi^ groups, adjusting for covariates including sex, cancer stage, and diagnosis age.

### IFNα bioassay

Following procedures previously described by Buzzai *et al.* (*16*), we measured bioactive IFNα using an in vitro IFN bioassay. Bioactive IFNα titers were determine by comparing the cytopathic effect of supernatants from B16.GFP.gB.luc, B16.IFNα4.gB.luc, and B16.IFNα9.gB.luc cell lines to the NIH IFNα standard (1000 IU/ml).

### IL-4 ELISA cell lines

Supernatants were collected from *Il4*-transduced B16 cell lines for quantification of IL-4 secretion. In brief, 2 × 10^5^ irradiated (20,000 rad) B16 cells in 2 ml of media were seeded per well of a 6-well plate, and the supernatant was collected 24 hours later. Cells were irradiated to account for any differences in cell proliferation between the cell lines. The supernatant was filtered through a 0.45-μm filter and stored at −80°C until use. The supernatant from control B16 cells were tested neat and at a 1:4 dilution, and the supernatant from *Il4*-transduced cells were tested at 1:8 and 1:32 dilutions. Analysis of IL-4 secretion by transduced B16 cells was performed using the LEGEND MAX Mouse IL-4 ELISA kit (BioLegend, USA) according to the manufacturer’s instructions. The plate was analyzed by measuring absorbance at 450 and 570 nm using a CLARIOstar plate reader (BMG LABTECH, Germany). Secreted IL-4 (ng/ml) was calculated by generating a standard curve, plotting the average absorbance (450 nm) obtained for each standard concentration. Values were first log-transformed and then fitted with a four-parameter logistic curve using GraphPad Prism software (version 8.3.1). The amount of IL-4 in each sample was calculated by interpolating from the standard curve and then multiplying by the dilution factor.

### IL-4 ELISA T cells

Supernatants were collected from *Il4*-transduced gBT.I cells for quantification of IL-4 secretion. In brief, 2 × 10^6^ T cells were plated in 1 ml of media were seeded per well of a 24-well plate, and the supernatant was collected 24 hours later. Cellular debris was removed by centrifugation, and the supernatant was collected and stored at −80°C until use. Analysis of IL-4 secretion by transduced and nontransduced gBT.I cells was performed using the Mouse IL-4 ELISA kit (Thermo Fisher Scientific, USA) according to the manufacturer’s instructions. The plate was analyzed by measuring absorbance at 450 and 570 nm using a CLARIOstar plate reader (BMG LABTECH, Germany). Secreted IL-4 (ng/ml) was calculated by generating a standard curve, plotting the average absorbance (450 nm) obtained for each standard concentration. Values were first log-transformed and then fitted with a four-parameter logistic curve using GraphPad Prism software (version 8.3.1). The amount of IL-4 in each sample was calculated by interpolating from the standard curve and then multiplying by the dilution factor.

### Bone marrow chimera generation

Drinking water of recipient WT and IFNAR^o/o^ mice was supplemented with neomycin sulfate (25 mg/ml) and polymyxin B sulfate (13 mg/liter). Two weeks later, recipient mice were irradiated twice at 550 rad (3 hours apart) and then intravenously injected with donor bone marrow cells. Briefly, to prepare bone marrow cells for transfer, donor WT or IFNAR^o/o^ mice were euthanized and bone marrow was collected from hind legs. After red blood cell lysis, bone marrow was filtered through a 190-μm metal mesh to generate a single-cell suspension. The single-cell suspension was treated with 10 μl of T cell depletion cocktail [anti-CD4 (GK1.5), anti-CD8 (53.6.7), and anti-Thy1.1 (T24/31.6) in RPMI] per 10^6^ cells for 30 min at 4°C. After incubation, the suspension was suspended in BioMag Goat Anti-Rat IgG magnetic beads (Qiagen, DE) at a 3:1 ratio and incubated for 20 min at 4°C. This suspension was then placed onto a magnet to remove T cells from the bone marrow suspension. The T cell–depleted bone marrow was washed 3x prior to injection of 10^6^ cells into lethally irradiated recipient mice. The following day, recipient mice were further depleted of T cells with intraperitoneal injection of 50 μg anti-Thy1.1 (T24/31.7). Chimeric mice were for 8 weeks posttransfer to allow for bone marrow reconstitution before subcutaneous tumor challenge.

### Subcloning pMICh-Il4

Coding sequence for murine *Il4* was ordered from IDT as g-blocks (Integrated DNA Technologies, Singapore) and polymerase chain reaction (PCR) amplified using the Phusion High Fidelity DNA polymerase kit (New England Biolabs, USA). The PCR-amplified fragment was purified using the Promega Wizard SV Gel and PCR cleanup system as per the manufacturer’s instructions. For cloning, 5 μg of the MSCV-IRES-mCherry plasmid (pMICh) was digested with *EcoRI* (New England Biolabs, USA). To prevent recombination of the linearized pMICh vector, 1 U of recombinant Shrimp Alkaline Phosphatase (New England Biolabs, USA) was used. Digested pMICh vector and digested and amplified inserts were purified using the Monarch PCR & DNA Cleanup Kit (New England Biolabs, USA) according to the manufacturer’s instructions. Ligation reactions were performed using a molar ratio of 1:3 (vector:insert). Ligation reactions were set up using T4 DNA ligase (New England Biolabs, USA) and left to incubate for 3 hours at room temperature before inactivating at 65°C for 10 min. One microliter of ligation reaction was added to competent DH5a *Escherichia coli* (New England Biolabs, USA) and heat shocked at 42°C for 30 s and then placed on ice for a further 3 min. Cells were incubated in 1 ml of SOC recovery buffer (New England Biolabs, USA) at 37°C for 1 hour, shaking at 250 rpm. The bacteria and SOC solution were spread onto lysogeny broth (LB)–ampicillin agar plates and incubated at 37°C overnight.

To generate small stocks of plasmid DNA, overnight cultures of transformed *E. coli* colonies were grown in LB with ampicillin (10 μg/ml) at 37°C, shaking at 250 rpm. Bacteria was harvested by centrifugation at 2500*g* for 5 min, and then plasmid DNA was extracted from bacterial pellet using the Wizard Plus SV Minipreps DNA Purification System (Promega, USA) as per the manufacturer’s instructions. Recombinant plasmid DNA was sent for Sanger sequencing at the Australian Genome Research Facility in Perth, Western Australia. Sequencing alignment was analyzed using SnapGene5.2.2 software (USA).

### Statistics

Statistical analyses for flow cytometry and in vivo experiments were performed using GraphPad (GraphPad Software Inc. v10.2.0). Comparison of cell populations was assessed using one-way or two-way ANOVA with a Tukey post hoc test or Šídák post hoc test, respectively. Difference in tumor survival was compared using the log-rank Mantel-Cox test, and tumor growth was measured by a repeated-measures two-way ANOVA (mixed-model) followed by Tukey post hoc test. Statistical significance is indicated as *P < 0.05, **P < 0.01, ***P < 0.005, and ****P < 0.001, unless otherwise stated.
