# Supplementary material for: Interleukin-4 modulates type I interferon to augment antitumor immunity
Source: Sci Adv. 2025 May 14;11(20):eadt3618. doi: 10.1126/sciadv.adt3618 (PMC12077506; doi:10.1126/sciadv.adt3618)
Supplement: Supplementary file 1 — Figs. S1 to S7 Legend for data S1 [file sciadv.adt3618_sm.pdf]

Science Advances
AAAS

Supplementary Materials for

Interleukin-4 modulates type I interferon to augment antitumor immunity

Hannah V. Newnes *et al.*

Corresponding author: Anthony Bosco, abosco@arizona.edu; Jason Waithman, jason.waithman@uwa.edu.au

Sci. Adv. **11**, eadt3618 (2025)
DOI: 10.1126/sciadv.adt3618

The PDF file includes:

Figs. S1 to S7
Legend for data S1

Other Supplementary Material for this manuscript includes the following:

Data S1

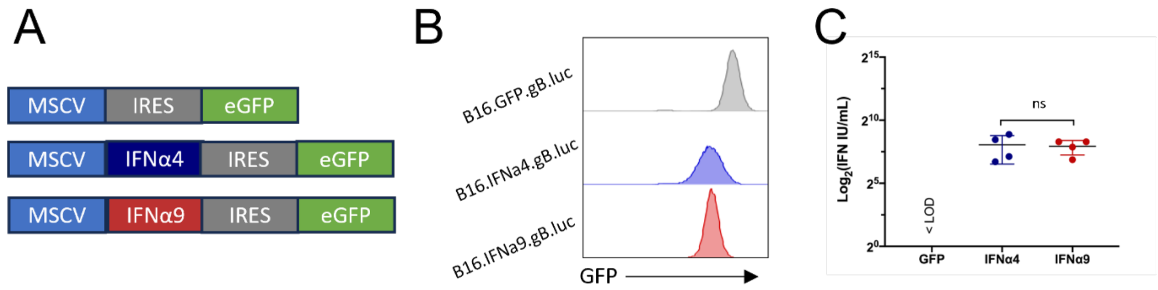

Fig. S1. Characterisation of transduced B16 cells which secrete individual IFNα subtypes. (**A**) B16.gB.luc cell lines were transduced with retrovirus containing the murine stem cell virus LTR promotor driving expression of an individual IFNα subtypes, internal ribosome entry site (IRES) and enhanced green fluorescent protein (eGFP). (**B**) Cell lines were analysed by flow cytometry for GFP fluorescence. (**C**) Supernatant was harvested from cell lines and diluted across individual wells of 96-well plate seeded with L929 cells. After 24 hours, the supernatant was replaced with media containing EMCV. Twenty-four hours later semi-quantitative measurement of the amount of IFNα produced by each cell line titrated against the IFNα international standard. Data was pooled from two independent experiments and analysed by a one-way ANOVA. LOD, limit of detection; IU, international units.

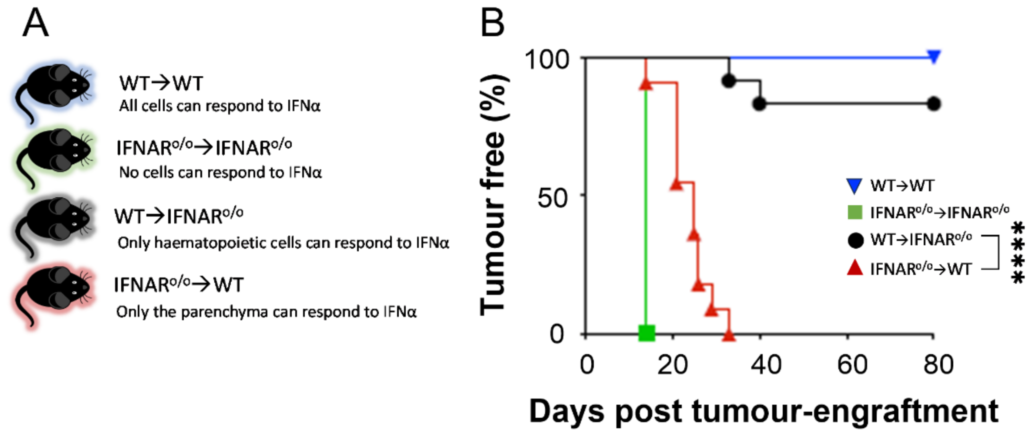

Fig. S2. Anti-tumour effect of IFNα9 depends on signalling through the haematopoietic compartment. (**A**) Chimeras were generated by intravenous transfer of 106 bone marrow cells derived from C57Bl/6 (WT) or IFNα receptor knock-out (IFNAR$^{o/o}$) mice into lethally irradiated WT or IFNAR$^{o/o}$ mice (n=5 per group for WT®WT and IFNAR$^{o/o}$→IFNAR$^{o/o}$, n=11-12 per group for WT→IFNAR$^{o/o}$ and IFNAR$^{o/o}$→WT). (**B**) After 8-weeks reconstitution, mice were challenged with 5x10^5 B16.IFNα9.gB.luc cells subcutaneously and monitored for tumour development. Data was pooled from two independent experiments and analysed by Log-Rank Mantel-Cox test. Significance reported as ****p<0.0001 (WT→IFNAR$^{o/o}$ vs IFNAR$^{o/o}$→WT).

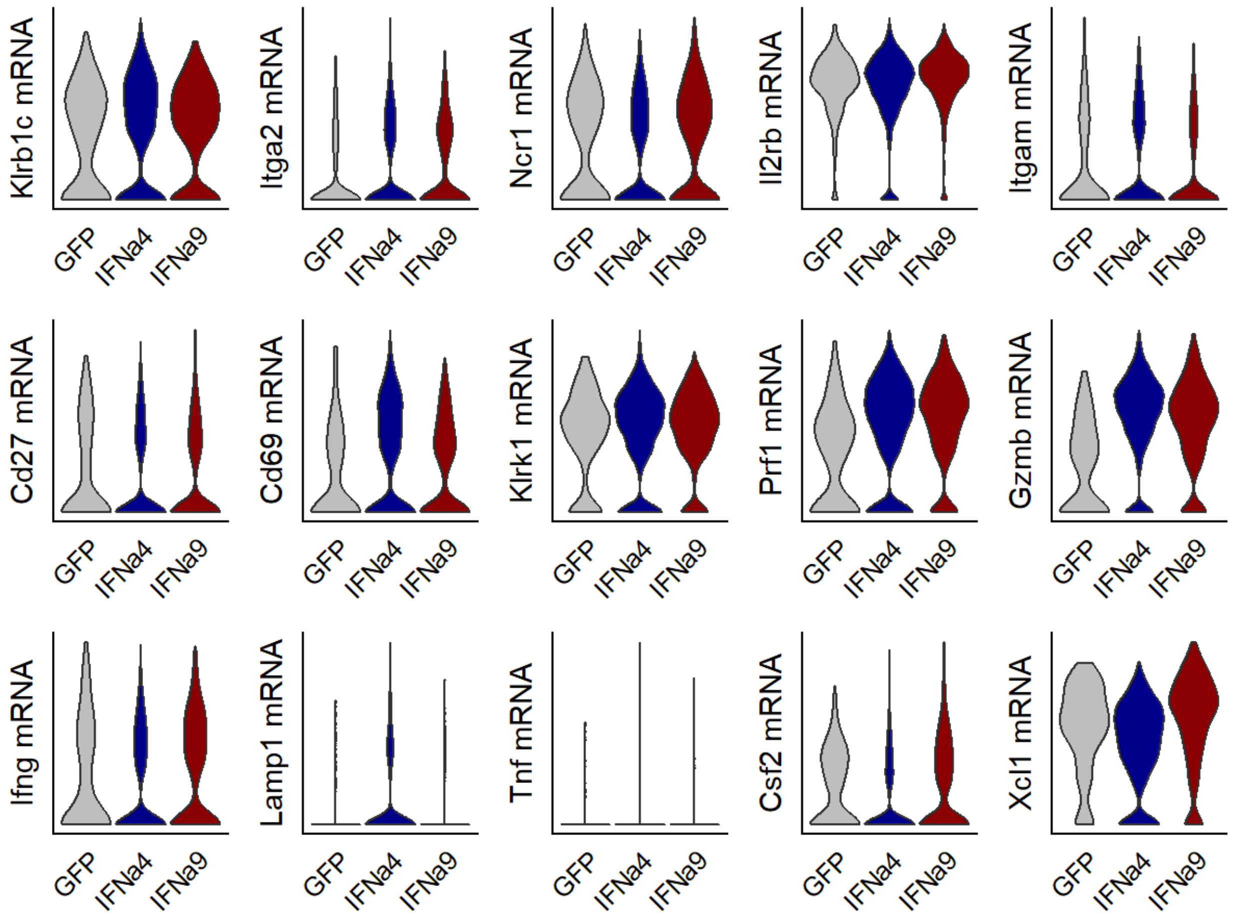

Fig. S3. Expression of NK cell (C02) activation and marker genes. Violin plots depicting the expression levels of transcripts encoding NK cell markers between B16.GFP.gB.luc, B16.IFNα4.gB.luc and B16.IFNα9.gB.luc tumours.

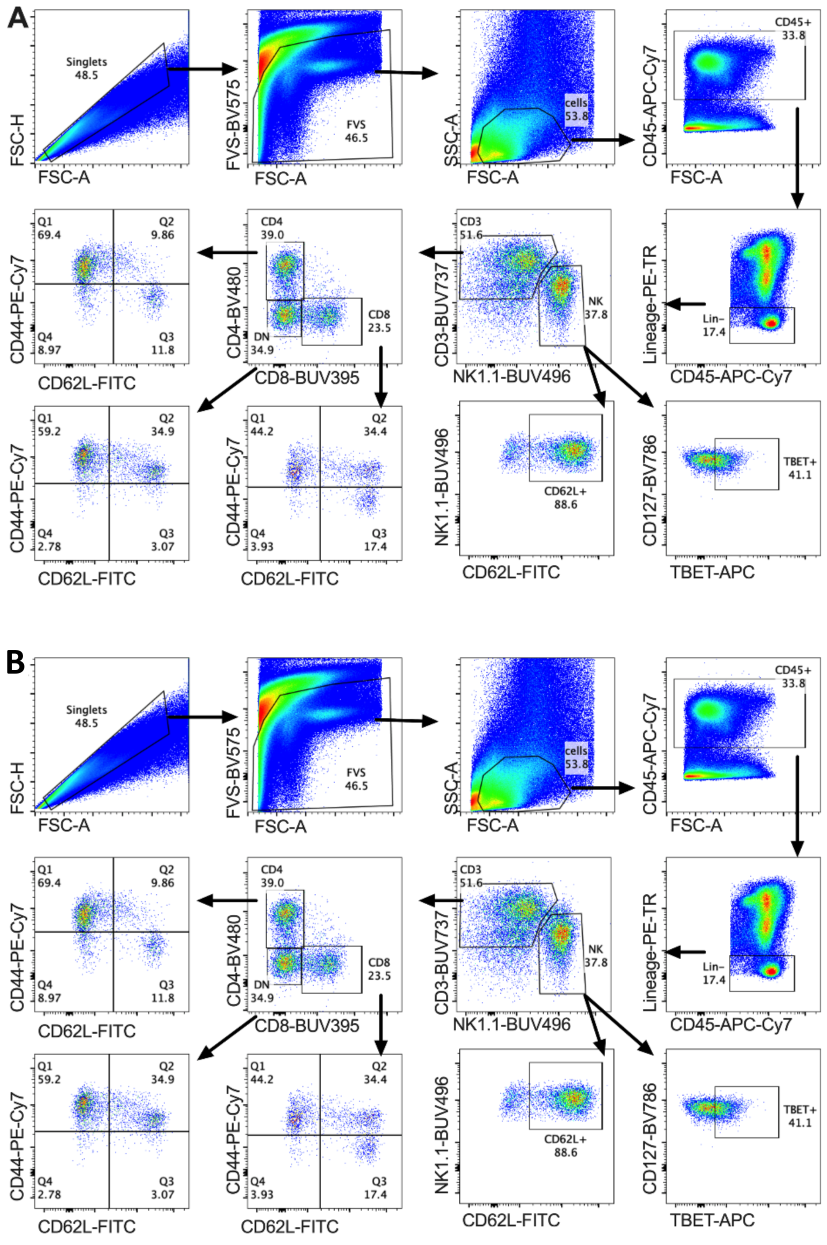

Fig. S4. Gating strategies to analyse cell population frequencies by flow cytometry. C57Bl/6 mice were challenged with $5x10^5$ B16.GFP.gB.luc, B16.IFNα4.gB.luc or B16.IFNα9.gB.luc cells. Eight days post engraftment tumours were harvested and analysed by flow cytometry. (**A**) Example gating strategy for analysis of key immune cell populations. (**B**) Example gating strategy for analysis of T and NK cell populations.

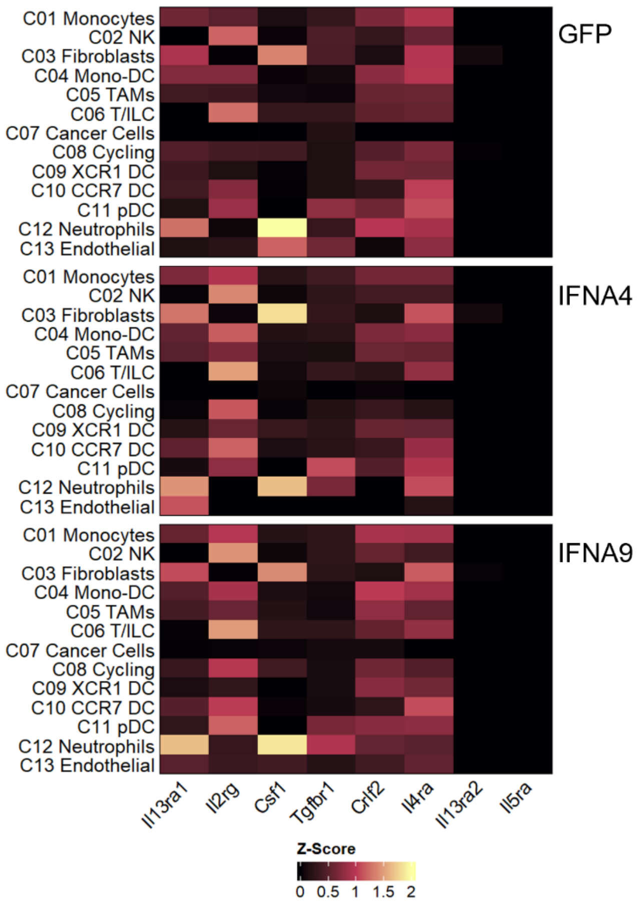

Fig. S5. Expression of receptor transcripts associated with Type-2 inflammation across each condition. Cluster-wise average gene expression levels (per condition) of transcripts encoding receptor subunits for Type-2 inflammatory ligands such as IL-4, TSLP, IL-13, M-CSF, TGFβ-1 and IL-5.

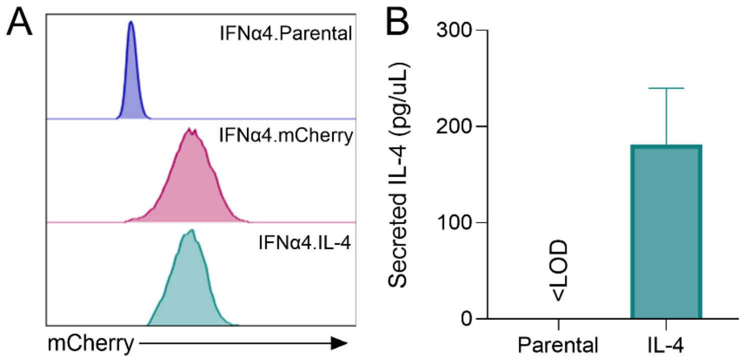

Fig. S6. Transduced B16.IFNα4.gB.luc cells secrete interleukin 4. (**A**) Representative histograms of mCherry expression from non-transduced and transduced B16.IFNa4.gB.luc cells. (**B**) Amount of secreted IL-4 (pg/µL) from transduced B16.IFNα4.gB.luc cells as measured by ELISA assay. Data was pooled from three biological repeats. Error bars indicate SD. <LOD, below the limit of detection.

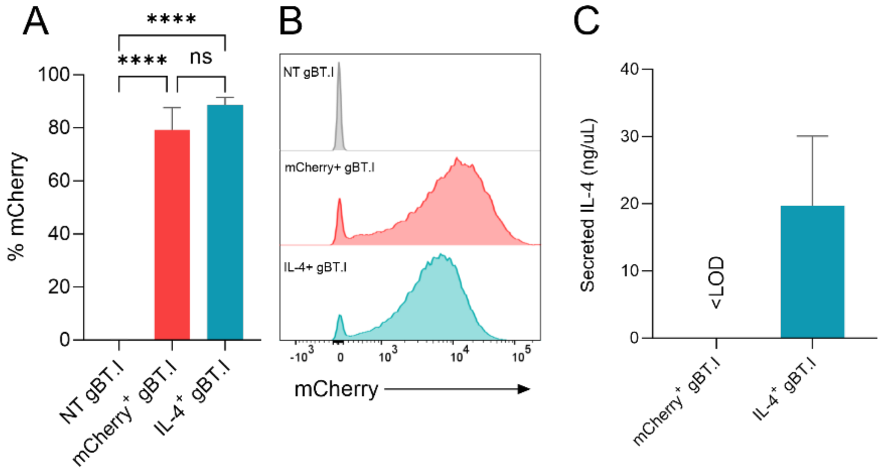

Fig. S7. **Transduced gBT.I cells secrete interleukin 4.** (**A**) Mean percentage of mCherry positive cells from non-transduced (NT) and transduced gBT.I cells. Data was pooled from three independent experiments; error bars indicate SD. Data was analysed by one-way ANOVA followed by a Tukey post hoc test. (**B**) Representative histograms. (**C**) Amount of secreted IL-4 (ng/µL) from transduced gBT.I cells as measured by ELISA assay. Data was pooled from three biological repeats. Error bars indicate SD. Significance reported as ****p<0.0001. <LOD, below the limit of detection.

Data S1. (separate file). Differentially expressed genes for each scRNA-seq cluster. Marker genes (for clusters C01-C13) were identified by comparing profiles for one cluster against all clusters in the dataset using the Findmarkers() function in the Seurat package. Marker genes (positively enriched) were calculated using a Wilcoxon Ranked Sum test (with Bonferroni correction for multiple comparisons).
